# Comparative evaluation of small molecules reported to be inhibitors of *Staphylococcus aureus* biofilm formation

**DOI:** 10.1128/spectrum.03147-23

**Published:** 2023-12-07

**Authors:** Mara J. Campbell, Karen E. Beenken, Horace J. Spencer, Bina Jayana, Hana Hester, Gyan S. Sahukhal, Mohamed O. Elasri, Mark S. Smeltzer

**Affiliations:** 1 Department of Microbiology and Immunology, University of Arkansas for Medical Sciences, Little Rock, Arkansas, USA; 2 Department of Biostatistics, University of Arkansas for Medical Sciences, Little Rock, Arkansas, USA; Icahn School of Medicine at Mount Sinai, New York, New York, USA

**Keywords:** *Staphylococcus aureus*, biofilms, infectious disease, antimicrobial agents, antibiotic resistance, antimicrobial activity

## Abstract

Biofilm formation is an important characteristic of many *Staphylococcus aureus* infections because it limits the efficacy of host defenses and conventional antibiotic therapy. There are many literature reports that describe small molecule inhibitors of *S. aureus* biofilm formation, but the lack of direct comparisons and differences in the methods used to assess inhibition make it impossible to assess the efficacy of these compounds relative to each other. To address this, we compared 19 compounds reported in the literature to be inhibitors of *S. aureus* biofilm formation. We used the methicillin-susceptible clinical osteomyelitis isolate UAMS-1 in a microtiter plate biofilm assay shown to maximize biofilm formation and provide results consistent with those observed *in vivo*. Under these conditions, telithromycin (Ketek) showed the greatest inhibitory activity, limiting biofilm formation to a degree comparable to a UAMS-1 *sarA* mutant at a concentration of 0.49 µM (0.40 µg/mL). Similar results were obtained for the methicillin-resistant USA300 strain LAC. Telithromycin is a bacteriostatic ketolide antibiotic that is not currently approved in the United States for use as an antibiotic due to its concerning safety profile. However, the concentration required to limit growth *in vitro* was higher than the concentration required to limit biofilm formation, suggesting that low-dose telithromycin might be useful to limit biofilm formation and increase the efficacy of other antibiotics in biofilm-associated infections. Irrespective of whether telithromycin is ultimately proven clinically useful in this regard, these results emphasize the need for widespread use of a standardized *in vitro* approach to evaluate prospective inhibitors.

**IMPORTANCE:**

Because biofilm formation is such a problematic feature of *Staphylococcus aureus* infections, much effort has been put into identifying biofilm inhibitors. However, the results observed with these compounds are often reported in isolation, and the methods used to assess biofilm formation vary between labs, making it impossible to assess relative efficacy and prioritize among these putative inhibitors for further study. The studies we report address this issue by directly comparing putative biofilm inhibitors using a consistent *in vitro* assay. This assay was previously shown to maximize biofilm formation, and the results observed with this assay have been proven to be relevant *in vivo*. Of the 19 compounds compared using this method, many had no impact on biofilm formation under these conditions. Indeed, only one proved effective at limiting biofilm formation without also inhibiting growth.

## INTRODUCTION

Biofilm formation is a defining characteristic of many forms of bacterial infection because it provides a therapeutically relevant level of intrinsic resistance to host defenses and conventional antibiotic therapy (1, 2). This is true of many, if not most, *Staphylococcus aureus* infections (3). Among the most clinically problematic of these are infections involving bone and indwelling medical devices including orthopedic implants. The correlation between biofilms and this intrinsic resistance accounts for the many reports focused on identifying inhibitors of *S. aureus* biofilm formation (1 – 3). Many of these reports described promising compounds identified in screens of small molecule libraries (4, 5), while others described studies with compounds that have already been explored for other clinical applications (6 – 10). Unfortunately, the methods used to assess biofilm formation are often inconsistent between research groups, making it impossible to draw conclusions about the relative inhibitory effect of these compounds.

We began to address this problem by making direct comparisons of commercially available reported inhibitors using an *in vitro* microtiter plate assay that maximizes *S. aureus* biofilm formation. Critical elements of this assay include pre-coating the substrate with human plasma and supplementing the media (tryptic soy broth) with both salt and glucose (11). While no *in vitro* condition can be assumed to fully represent *in vivo* reality, we have previously demonstrated the relevance of this assay in studies that demonstrated that the mutation of the staphylococcal accessory regulator (*sarA*) limits the biofilm formation *in vitro* (12) and *in vivo* to a degree that can be correlated with increased antibiotic susceptibility in a catheter-associated murine biofilm infection model, sepsis, and osteomyelitis (12 – 16). Moreover, these results were consistent across strains and diverse clinical isolates of *S. aureus* including methicillin-susceptible and methicillin-resistant strains (12 – 16), and mutations in regulatory genes that have little impact on biofilm formation in this assay also have little impact on virulence in our murine osteomyelitis model (17 – 19). These collective observations support our belief that the results we observe with our *in vitro* biofilm assay provide a reliable indicator of *in vivo* relevance and also suggest that *sarA* is a key regulatory target for the development of *S. aureus* biofilm inhibitors. Indeed, based on the relationship between *sarA* and biofilm formation, many reports specifically attempted to identify compounds that act as inhibitors of *sarA* (5, 20 – 22), and to the extent possible, these were prioritized for inclusion in the comparisons we report.

Accordingly, we employed our microtiter plate assay to assess the impact of 19 compounds reported to be inhibitors of *S. aureus* biofilm formation relative to each other. We compared the results observed with each compound to those observed with a *sarA* mutant, which serves as a control demonstrating a level of biofilm inhibition that is known to be clinically relevant. Initial comparisons were done with the methicillin-susceptible USA200 strain UAMS-1, which was isolated from the bone of an osteomyelitis patient during surgical debridement and is known to form a robust biofilm (11 – 16). Studies with the two compounds found to inhibit biofilm formation to the greatest extent, specifically celastrol and telithromycin, were then extended to include the methicillin-resistant USA300 strain LAC.

## RESULTS

### Relative impact on individual compounds on biofilm formation

Nineteen commercially available compounds reported to be inhibitors of *S. aureus* biofilm formation were identified from the literature and evaluated at concentrations ranging from 250 to 0.015 µM. Of these 19 compounds, seven had no impact on biofilm formation with UAMS-1 even at a concentration of 250 µM (Table 1). These compounds were erianin, 2-[(methylamino)methyl]phenol, SarABI^M^/UTI^QQ^, lapatinib (Tykerb), taxifolin, 3-hydroxybenzoic acid, and hibifolin. One (alizarin) inhibited biofilm formation to a statistically significant degree relative to UAMS-1, but only at a concentration of 125 µM, and it did not result in a reduction of biofilm formation comparable to the UAMS-1 *sarA* mutant at even 250 µM. Eleven other compounds inhibited biofilm formation by comparison to UAMS-1 at concentrations ranging from 0.49 µM (telithromycin) to 250 µM (clemastine), with most doing so in the range of 62.5–125 µM (Table 1). The three compounds that inhibited biofilm formation to the greatest extent at the lowest concentrations were aloe-emodin, celastrol, and telithromycin, which inhibited biofilm formation to a statistically significant degree by comparison to UAMS-1 at concentrations of 3.91, 3.91, and 0.12 µM, respectively. All three of these compounds also inhibited biofilm formation to a degree comparable to that observed in the UAMS-1 *sarA* mutant, but aloe-emodin only did so at concentrations ≥62.5 µM. Celastrol and telithromycin, on the other hand, inhibited biofilm formation in UAMS-1 to a degree comparable to that observed in its isogenic *sarA* mutant at concentrations of 3.91 and 0.49 µM, respectively (Table 1).

**TABLE 1:** Summary of reported biofilm inhibitor original test conditions and efficacy in a standardized assay^*g*^

Compound	Reference	Biofilm assay conditions	Strain	Different than UAMS-1^*a*^	Same as Δ*sarA^b^*
Erianin^*c*^	(23)	BHI, 3% sucrose	Newman	>250 µM	>250 µM
2-[(Methylamino)methyl]phenol^*d*^	(20)	TSB, 0.5% glucose	Clinical isolates P1966 and AB459	>250 µM	>250 µM
SarABI^M^ (UTI^QQ^)^*e*^	(24)	Artificial urine media	ATCC 25923	>250 µM	>250 µM
Lapatinib^*c*^	(6)	TSB, 0.5% glucose	ATCC 29213, SA113, FPR3757	>250 µM	>250 µM
Taxifolin^*c*^	(8)	BHI, 0.5% glucose, 3% NaCl	USA300^*c*^	>250 µM	>250 µM
3-Hydroxybenzoic acid^*c*^	(21)	TSB	SA-01, SA-02	>250 µM	>250 µM
Hibifolin^*c*^	(25)	BHI, 0.5% glucose, 3% NaCl, 20% freeze-dried rabbit blood coating	USA300	>250 µM	>250 µM
Alizarin^*c*^	(26)	LB^*d*^	ATCC 6538, ATCC 25923, MW2	125 µM	>250 µM
Clemastine^*c*^	(7)	TSB, 0.5% glucose	Multiple clinical isolates	250 µM	250 µM
Hypericin^*c*^	(22)	BHI, 0.5% glucose	JE2	125 µM	250 µM
Loratadine^*c*^	(10)	TSB, 0.5% glucose	ATCC 29213 and 35556	125 µM	250 µM
Kaempferol^*c*^	(27)	BHI, 5% glucose/3% NaCl, 20% rabbit plasma	ATCC 29213	62.5 µM	125 µM
Boeravinone B^*c*^	(28)	TSB^*b*^, 2% glucose	SA1199	7.81 µM	125 µM
Efavirenz^*c*^	(8)	TSB, 0.5% glucose`	Multiple clinical isolates	62.5 µM	62.5 µM
Ticagrelor^*c*^	(29)	LB, 0.5% glucose	Multiple clinical isolates	62.5 µM	62.5 µM
Zinc00990144^*f*^	(5)	TSB, 0.5% glucose	Multiple clinical isolates	15.63 µM	62.5 µM
Aloe-emodin^*c*^	(30)	BHI^*a*^, 5% glucose/3% NaCl, 20% rabbit plasma	ATCC 29213	3.91 µM	62.5 µM
Celastrol^*c*^	(9)	BHI, 0.5% glucose	ATCC 6538	3.91 µM	7.82 µM
Telithromycin^*c*^	(31)	TSB, 2.5% glucose	Multiple clinical isolates, ATCC 29213	0.12 µM	0.49 µM

^*a*^ Indicates the concentration required to limit biofilm formation to a statistically significant degree by comparison to UAMS-1.

^*b*^ Indicates the concentration required to limit biofilm formation to a degree that was not statistically significant by comparison to the results observed with the sarA mutant.

^*c*^ Sigma-Aldrich.

^*d*^ TCI Chemicals.

^*e*^ Chemspace.

^*f*^ Specs.

^*g*^ Nineteen commercially available compounds were identified from the literature as reported biofilm inhibitors. The reference for the original reports and their assay conditions/strains are listed, as well as the concentrations necessary to significantly alter biofilm formation in comparison to the parent strain in the absence of any putative inhibitor, and the concentration necessary to reduce biofilm formation to the same degree as a *sarA* mutant in a standardized biofilm inhibition assay utilizing plasma pre-coating and tryptic soy broth supplemented with 0.5% glucose and 3% NaCl.

### Impact of celastrol on biofilm formation and growth *in vitro*

**Fig 1:**
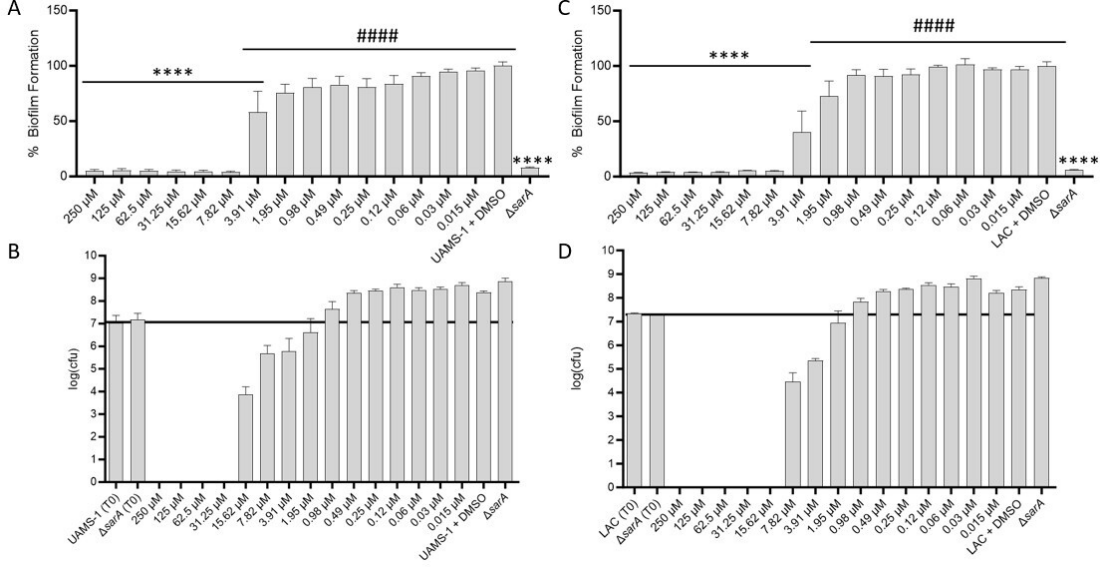
Inhibition of biofilm formation by celastrol requires concentrations that also inhibit growth. Biofilm formation and growth inhibition were assessed for UAMS-1 (**A, B**) and LAC (**C, D**) with the designated concentrations of celastrol. Biofilm formation is presented as the percent of biofilm relative to the dimethylsulfoxide (DMSO) control without celastrol. Significance was assessed by one-way analysis of variance (ANOVA) with Dunnett’s correction, where **** indicates *P* > 0.0001 against the DMSO control and #### indicates significance against the isogenic *sarA* mutant (Δ*sarA*). Growth in the presence of celastrol is presented as log_10_(colony-forming units) [log(CFU)], where bars above the black line indicate growth above the log(CFU) at the time of inoculation (**T0**) and bars at or below the black line indicate a lack of growth.

To confirm these results and determine whether the inhibition of biofilm formation could be attributed to reduced growth, we repeated our experiments with these two compounds and included independent assays to assess biofilm formation and growth in planktonic culture in the presence of the same concentration of each compound. The results confirmed that the concentration of celastrol required to limit biofilm formation to a statistically significant degree by comparison to UAMS-1 was 3.91 µM (1.78 µg/mL), while the concentration required to limit biofilm formation to a degree comparable to that observed with the UAMS-1 *sarA* mutant was 7.81 µM (3.52 µg/mL) (Fig. 1). However, these same concentrations were also found to limit the growth of UAMS-1. In fact, celastrol appeared to limit bacterial viability at concentrations >1.95 µM. In the methicillin-resistant USA300 strain LAC, the concentration required to limit biofilm formation by comparison to the parent strain was 3.91 µM (1.78 µg/mL), and this concentration was also found to limit biofilm formation to a degree that was statistically comparable to a LAC *sarA* mutant (Fig. 1). However, as with UAMS-1, this concentration was also found to limit the growth and viability of LAC in biofilm medium (BFM).

### Impact of telithromycin on biofilm formation and growth *in vitro*

**Fig 2:**
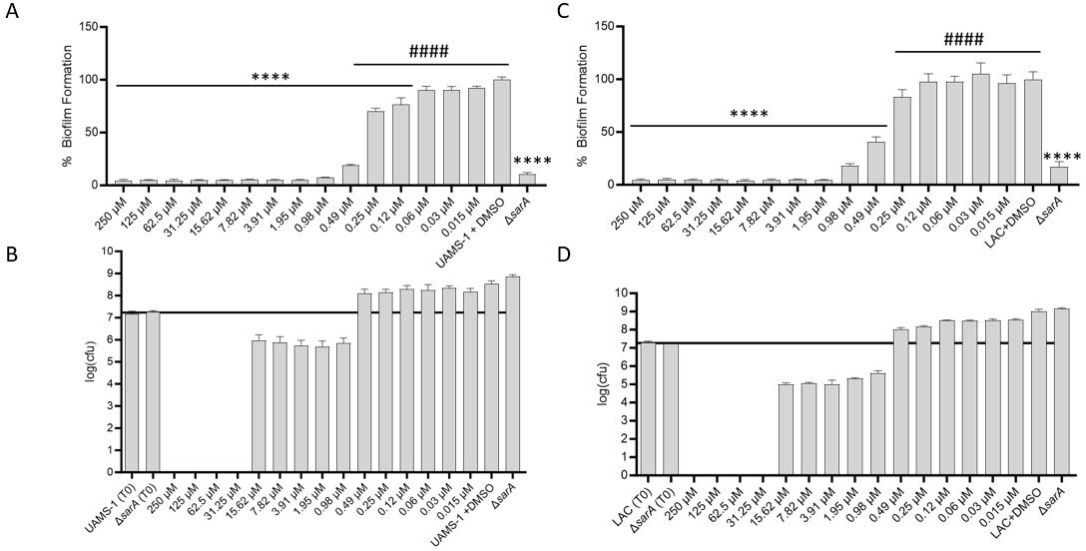
Telithromycin can inhibit biofilm formation at sub–minimum inhibitory concentrations (MICs). Biofilm formation and growth inhibition were assessed for UAMS-1 (**A, B**) and LAC (**C, D**) with the designated concentrations of telithromycin. Biofilm formation is presented as the percent (%) of biofilm mass relative to the telithromycin untreated DMSO-only control. Significance was assessed by one-way ANOVA with Dunnett’s correction, where **** indicates *P* > 0.0001 against the DMSO control and #### indicates significance against the isogenic *sarA* mutant (Δ*sarA*). Growth in the presence of telithromycin is presented as log_10_(colony-forming units) [log(CFU)], where bars above the black line indicate growth by comparison to the log(CFU) at T0 and bars at or below the black line indicate a lack of growth or reduction in cell viability.

With telithromycin, 0.12 µM (0.10 µg/mL) and 0.49 µM (0.40 µg/mL) were sufficient to limit biofilm formation by comparison to UAMS-1 and LAC, respectively (Fig. 2). A concentration of 0.49 µM was sufficient to limit biofilm formation to a degree comparable to an isogenic *sarA* mutant in both strains. Unlike celastrol, which did not inhibit biofilm formation at any concentration that did not also limit growth, 0.49 µM telithromycin did not inhibit the growth of LAC or UAMS-1 (Fig. 2). However, at concentrations greater than 0.49 µM, telithromycin did limit bacterial viability in both UAMS-1 (Fig. 2B) and LAC (Fig. 2D).

### Impact of telithromycin on *sarA*-mediated regulation

**Fig 3:**
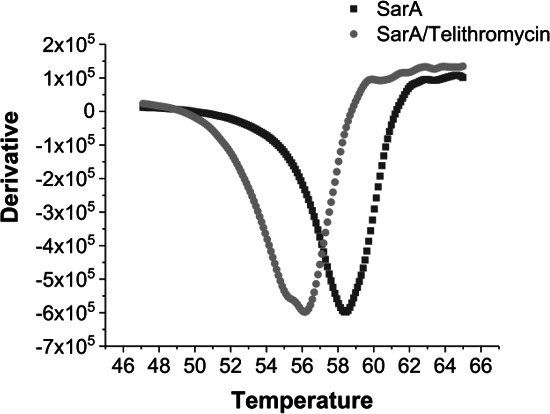
Thermal shift assay with telithromycin and purified SarA. Glomelt dye was used to determine the thermal stability of SarA in the presence of telithromycin. Analysis of melting temperature (Tm) was performed using the Boltzmann method from a plot of fluorescence intensity versus temperature.

When evaluating compounds for their ability to inhibit *S. aureus* biofilm formation, we made comparisons to the results observed with the wild-type strain and the isogenic *sarA* mutants. The *sarA* mutants were included as controls because we have demonstrated that the reduced capacity of *sarA* mutants to form a biofilm is correlated with increased antibiotic susceptibility and reduced virulence in biofilm-associated infections including osteomyelitis (11, 12, 14 – 16, 18, 19, 32, 33). Thus, the inclusion of *sarA* mutants in our studies was meant to provide an indication of potential clinical relevance rather than imply anything about the mechanism of action. However, there is a report concluding that the transcription of *sarA* is increased approximately twofold in post-exponential and stationary phase cultures in the presence of telithromycin (31). Because the mutation of *sarA* limits biofilm formation, such an increase would be expected to increase rather than decrease the capacity of *S. aureus* to form a biofilm. For the reasons discussed above, we used different assay conditions than most other reports, so to determine whether telithromycin impacts the transcription of *sarA* under our assay conditions, we examined the expression levels of *sarA* in the presence of 0.49-µM telithromycin in BFM. We found that the transcription of *sarA* was increased 2.721 ± 0.029- and 2.743 ± 0.034-fold in UAMS-1 and LAC, respectively (Table 2). This is consistent with the previous report, but as noted above, an increase in *sarA* transcription would not be expected to limit biofilm formation. At the same time, we demonstrated using a thermal shift assay that telithromycin binds SarA and reduces its thermal stability from 58.46°C to 55.69°C (Fig. 3). Although the concentration used in these experiments (4 mM) was higher than those used in our biofilm assays, this suggests that the effect of telithromycin on biofilm formation could be due to the inhibition of SarA function rather than its production.

**TABLE 2:** Gene expression results indicate that *sarA* transcription is increased in telithromycin-treated cells^*a*^

	*sarA* expression (fold change ± standard error)	
Strain	Celastrol	Telithromycin
UAMS-1	1.257 ± 0.101	2.721 ± 0.029
LAC	1.114 ± 0.03	2.743 ± 0.034

^*a*^ Cells were cultured in biofilm media with or without 0.49 μM of the indicated treatment. Relative change in the expression of *sarA* was calculated using *gyrA* as a control.

**Fig 4:**
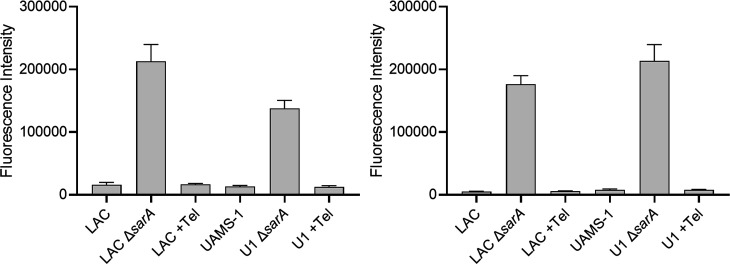
Impact of telithromycin on protease production. Overall protease activity was assessed using CM from overnight cultures of LAC and UAMS-1 with and without 0.49-µM telithromycin. CM from an overnight culture of the isogenic *sarA* mutant grown without telithromycin was included as a control. Results are shown after 1-h (left) and 24-h incubation of the EnzChek gelatinase/collagenase protease assay.

**Fig 5:**
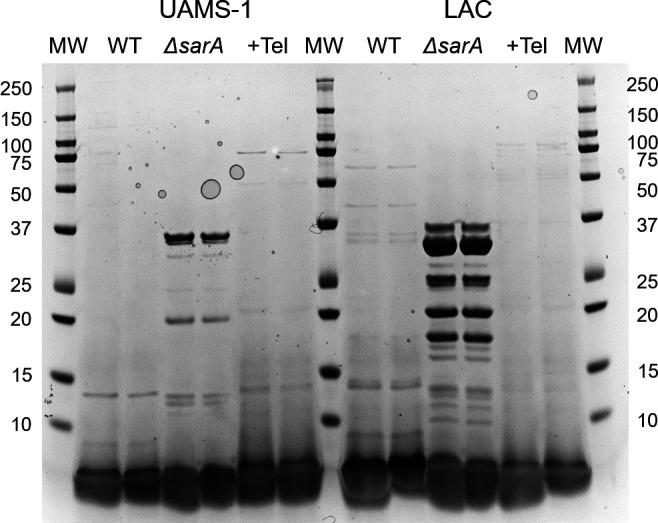
Impact of telithromycin on extracellular protein profiles. CM from duplicate cultures of UAMS-1 and LAC grown overnight with and without 0.49-µM telithromycin was examined by SDS-PAGE. MW indicates molecular size markers in kDa as shown.

To experimentally address this possibility, we took advantage of our demonstration that the reduced capacity of *sarA* mutants to form a biofilm can be attributed to the increased production of extracellular proteases (11, 34, 35). Specifically, we grew LAC and UAMS-1 in the presence of 0.49-µM telithromycin and assessed overall protease production. As expected, protease activity was significantly increased in both LAC and UAMS-1 *sarA* mutants, but it was not increased in the presence of telithromycin in either strain (Fig. 4). Similarly, the abundance of high-molecular-weight proteins was diminished in the conditioned medium (CM) from both *sarA* mutants, but not in CM from either parent strain grown in the presence of telithromycin (Fig. 5). This suggests that the impact of telithromycin is likely to be independent of *sarA*-mediated protease regulation.

**Fig 6:**
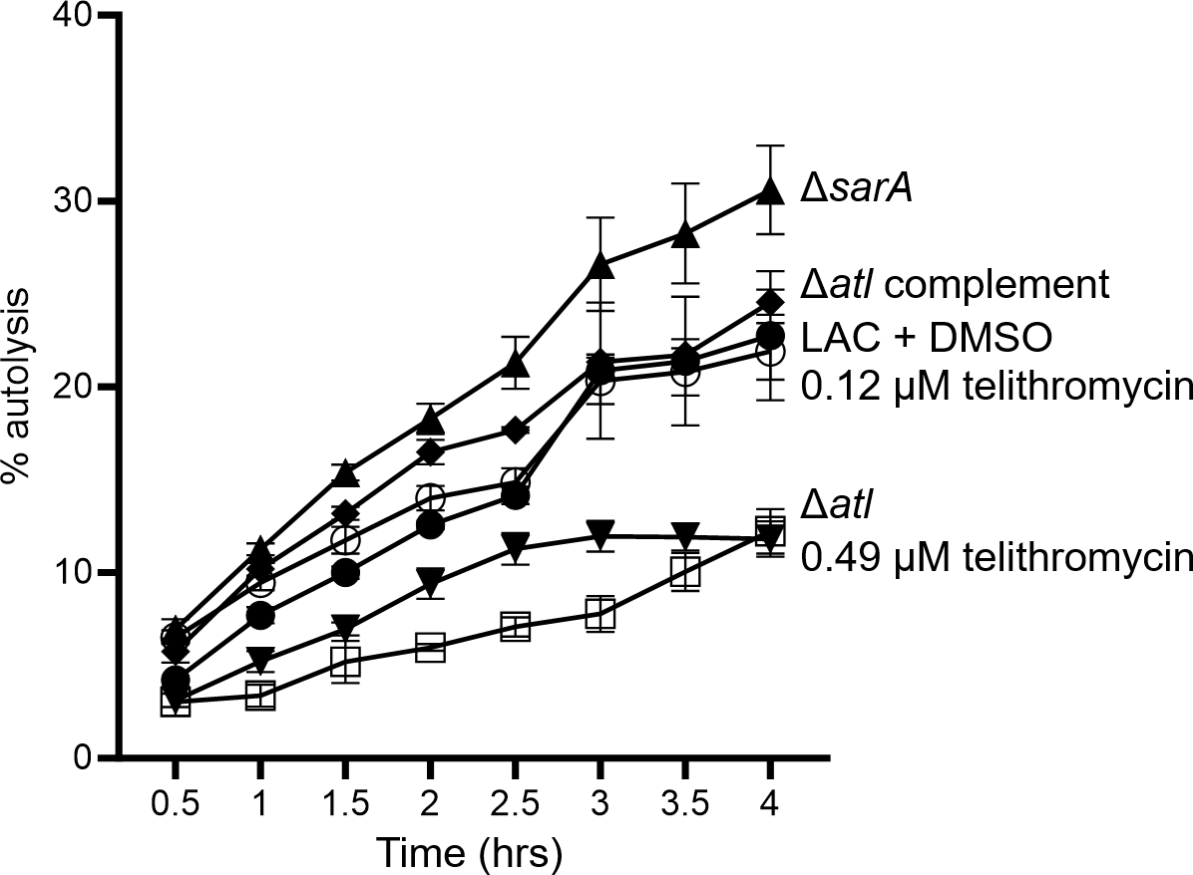
Impact of telithromycin on autolysis activity. Autolysis was assessed with the indicated mutants and conditions for LAC. The results are representative of two independent biological replicates with at least three experimental replicates each and presented as the percent (%) of autolysis at 30-min intervals for 4 h.

Telithromycin did not have an impact on protease production or overall SDS-PAGE profiles, but the mutation of *sarA* also impacts other phenotypes including autolytic activity, which is increased in a *sarA* mutant (36). Autolysin activity has also been correlated with biofilm-associated phenotypes including the abundance of eDNA (37) and cell wall anchoring of surface proteins including the fibronectin-binding proteins and protein A (38). Indeed, we found that 0.49-µM telithromycin did limit autolytic activity to a level comparable to an *atl* mutant (Fig. 6). In contrast, 0.12-µM telithromycin, a concentration that was not associated with reduced biofilm formation, did not result in increased autolysis.

### Impact of telithromycin on an established biofilm

**Fig 7:**
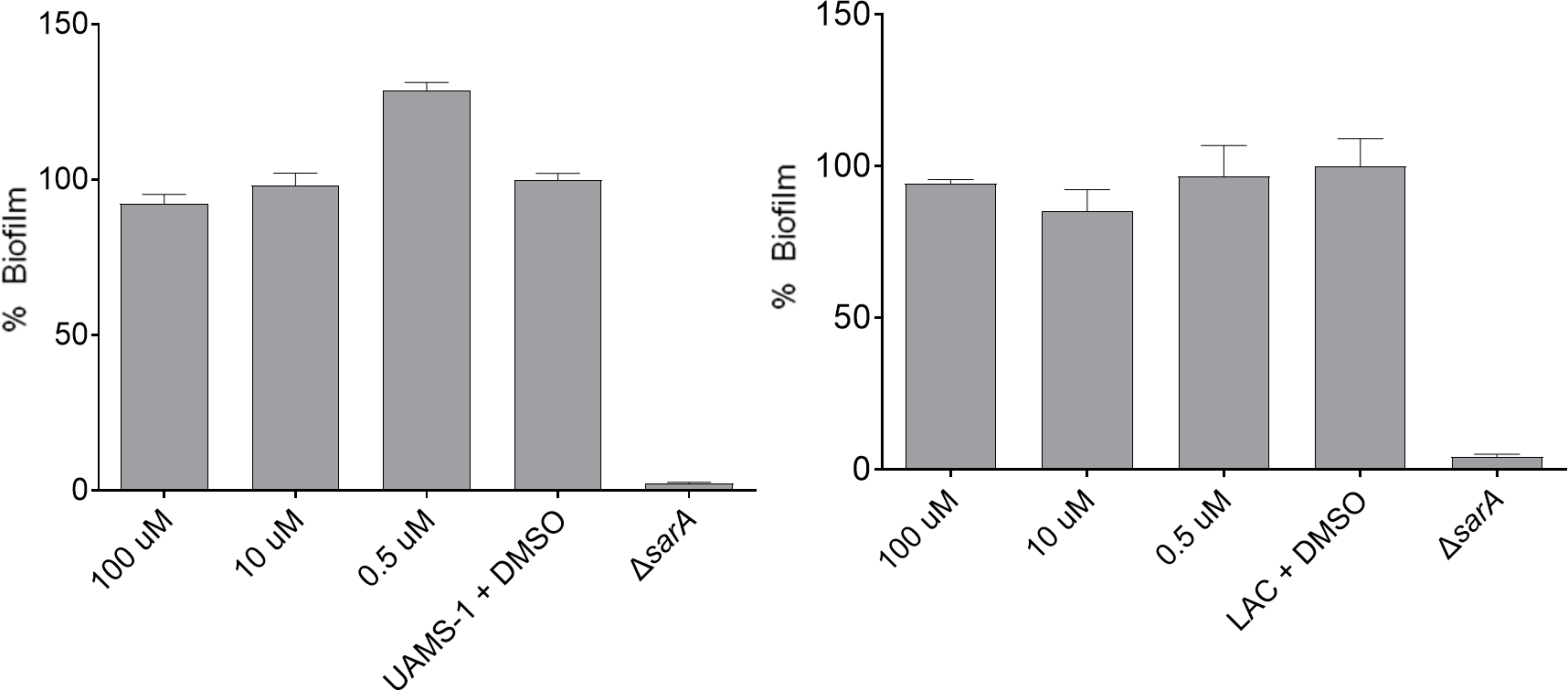
Impact of telithromycin on an established biofilm. Biofilms were allowed to form under our standardized experimental conditions before removing media and non-adherent cells and adding media containing the indicated amount of telithromycin in DMSO. The parent strain assayed with media containing an equivalent concentration of DMSO without telithromycin and the isogenic *sarA* mutant assayed with media that did not contain DMSO or telithromycin were included as controls. Results are reported as the percent (%) of biofilm mass remaining relative to the parent strain.

These studies suggest that telithromycin inhibits biofilm formation at a sub-inhibitory concentration, but they do not address whether it also has an impact in the context of an established biofilm. This is an important consideration with respect to the potential for therapeutic as well as prophylactic use. However, we were unable to demonstrate any reduction in biofilm mass in UAMS-1 or LAC after overnight exposure to telithromycin at a concentration as high as 100 µM, which, as noted above, is sufficient to limit the growth of both strains (Fig. 7).

## DISCUSSION

The clinical importance of biofilms is evident in the number of reports describing potential inhibitors of biofilm formation. However, differences in methodology make it impossible to draw conclusions about the relative efficacy of these inhibitors. Many recent reviews have emphasized the need for such inhibitors, discussed alternative strategies, and summarized compounds investigated in this context in diverse bacterial species (2, 3, 39 – 41). We have focused our efforts on *S. aureus* owing to its prominence as a cause of orthopedic infections including those involving bone and indwelling hardware, both of which are characterized to a significant extent by the formation of a biofilm (42 – 48). Biofilm formation and maintenance of the biofilm lifestyle in *S. aureus* are dynamic and complex processes influenced by many regulatory loci, but our studies have led us to focus on *sarA* because the mutation of *sarA* limits biofilm formation to a greater extent than mutation of any other regulatory locus we have examined, and it does so in diverse clinical isolates including methicillin-susceptible and methicillin-resistant strains (11 – 16, 18, 19, 34, 35, 49 – 51). Moreover, these previous studies confirmed that this can be correlated with increased antibiotic susceptibility and reduced virulence in animal models of osteomyelitis and implant-associated infection.

Thus, we undertook the current studies to make direct comparisons of inhibitors of *S. aureus* biofilm formation including putative inhibitors of *sarA* regulatory functions. Given the large number of reported inhibitors, it was not possible to be as fully comprehensive as we would have liked. However, we did identify 19 compounds that reportedly inhibited *S. aureus* biofilm formation and were readily available from a commercial source, and we made direct comparisons between these 19 compounds. This was done using a microtiter plate biofilm assay that maximizes biofilm formation under *in vitro* conditions and has been shown to accurately reflect *in vivo* relevance (11 – 19, 34, 35, 49 – 51). Strains included in our experiments were the methicillin-susceptible strain UAMS-1 and the methicillin-resistant strain LAC along with their isogenic *sarA* mutants as controls. Under these conditions, the two compounds that limited biofilm formation by comparison to both LAC and UAMS-1 at the lowest concentrations and did so to a degree comparable to their isogenic *sarA* mutants were celastrol and telithromycin.

Celastrol has been under investigation as a potential therapeutic in the context of a number of diseases including rheumatoid arthritis and cancer (52 – 54). It is a triterpene derived from the root of what is known as the Thunder God Vine (*Tripterygium wilfordii*), which has a long history of use in traditional Chinese medicine and is currently available as a nutritional supplement (52 – 54). However, according to the National Center for Complementary and Integrative Health, the many risks and side effects of Thunder God Vine extract are likely to outweigh its benefits (https://www.nccih.nih.gov/health/thunder-god-vine). Nevertheless, in the context of *S. aureus* infection, Yehia et al. (9) concluded that celastrol has several important properties including limiting the production of staphyloxanthin, increasing sensitivity to hydrogen peroxide, decreasing cell membrane integrity, decreasing survival in whole blood, and limiting biofilm formation (9). However, using our biofilm assay, we were unable to identify any concentration of celastrol that limited biofilm formation in UAMS-1 or LAC without also significantly limiting growth.

The concentration of celastrol that limited growth in comparison to the colony counts obtained immediately after inoculation (T0) was approximately 1.95 µM (0.89 µg/mL). The reported minimum inhibitory concentration of celastrol is 1 µg/mL (2.2 µM) as defined for the *S. aureus* strain ATCC 6538 (9). Another report found that the MIC of the methicillin-susceptible strain ATCC 29213 and a methicillin-resistant strain isolated from an osteomyelitis patient was 2 µg/mL (4). Our results are generally consistent with these previous reports, although it is not possible to make direct comparisons because the previous authors used different strains of *S. aureus* and did not use BFM as the test medium to determine MIC. While BFM is not normally used to determine MIC values, we believe it is appropriate in this case because it is consistent with the growth conditions used in our biofilm inhibition assays, and we are confident that the *in vivo* relevance of these *in vitro* conditions has been validated. Thus, we conclude from these results that the inhibition observed with celastrol in our biofilm assay is attributable to reduced growth.

Interestingly, a previous report found that when used at levels that did not limit cell viability or metabolic activity (≤440 nM), celastrol was associated with decreased expression of *icaA*, *msaB*, *sarA*, and *sigB* and increased expression of *agrA* and *icaR* (9), all of which have been correlated with a biofilm-deficient phenotype (10, 12, 17, 55 – 58). Based on this report, we examined *sarA* gene expression in the presence of 0.49 µM (490 nM) of celastrol as we did for telithromycin. This concentration did not inhibit growth, but it also did not inhibit biofilm formation in either UAMS-1 or LAC (Fig. 1). Moreover, we were unable to demonstrate any significant change in the *sarA* expression levels (Table 2). Based on these results and its questionable safety profile, we do not believe celastrol is a viable candidate for use as an anti-biofilm agent in *S. aureus*.

In contrast, the concentrations of telithromycin required to limit biofilm formation in comparison to UAMS-1 and LAC (0.12 and 0.49 µM, respectively) and perhaps even more importantly to a level comparable to their isogenic *sarA* mutants (0.49 µM) were below the concentration that limited growth (0.98 µM). Concentrations of 0.12 and 0.49 µM correspond to 0.10 and 0.40 µg/mL, which are both below the previously reported MIC of 1.0–2.0 µg/mL for telithromycin and *S. aureus* (31). Telithromycin is a protein synthesis inhibitor marketed under the trade name Ketek, but it is not currently available in the United States owing to safety concerns including visual impairment and liver toxicity (59). Nevertheless, because this safety profile is based on its use as an antibiotic and because telithromycin limits biofilm formation at a lower concentration than required to limit growth much less have a bactericidal effect, it is possible that it could be used as an adjunct agent to limit biofilm formation and enhance susceptibility to other antibiotics while avoiding these toxicity issues. Moreover, we did not examine whether other more tolerable protein synthesis inhibitors might have the same inhibitory impact on biofilm formation.

The observation that telithromycin inhibited biofilm formation but had no impact on an established biofilm suggests that its utility would be limited to prophylactic use with little therapeutic utility in the context of an established biofilm-associated infection. However, it is important to note that traumatic injury to the bone involving penetration of the skin invariably results in a contaminated bone defect, thus necessitating surgical repair and concomitant measures to prevent infection. These measures often include local antibiotic delivery (43, 60, 61), and it is in this context that telithromycin combined with other antibiotics might be useful. Telithromycin has also been shown to have good bone penetration (62) and relatively little toxicity for human osteoblasts, at least by comparison to celastrol (9).

From a mechanistic point of view, while thermal shift assays suggested that telithromycin binds SarA, these assays were done *in vitro* using a concentration of telithromycin (4 mM) that greatly exceeded the concentration required to inhibit growth. At concentrations that did not inhibit growth but did limit biofilm formation, growth in the presence of telithromycin did not mimic primary protease-associated phenotypes that are characteristic of *sarA* mutants. Specifically, we did not observe an increase in the overall production of extracellular proteases or the degradation of *S. aureus* extracellular proteins, suggesting that the ability of telithromycin to inhibit biofilm formation is not mediated through SarA or any other regulatory proteins that significantly impact protease production. However, we did find that at a concentration correlated with reduced biofilm formation, telithromycin did limit autolytic activity and that autolytic activity was not limited at a lower concentration that also did not inhibit biofilm formation. It is difficult to investigate whether this effect on autolysis can be directly demonstrated to result in the reduced capacity to form a biofilm, because at the concentration found to limit autolytic activity, telithromycin also significantly limited biofilm formation, thus precluding the ability to quantify biofilm-associated eDNA or surface proteins. Thus, as of now, the specific mechanism for telithromycin’s inhibition of biofilm formation remains unclear. Irrespective of the mechanism involved, the results we present suggest that telithromycin may have prophylactic and perhaps even therapeutic value in biofilm-associated *S. aureus* infections, but this remains to be empirically determined.

The compounds evaluated here were chosen based on reports from the literature and their availability, and our studies were by no means comprehensive. Indeed, a PubMed search carried out during the preparation of this manuscript using the search terms “biofilm,” “inhibitor,” and “*Staphylococcus aureus*” identified 525 publications, many of which describe compounds that were generated *de novo* and are not readily available. As an example, a recent report described the synthesis of a compound designated CY-158-11 that limited biofilm formation in multiple *S. aureus* strains at a concentration as low as 0.125 µg/mL (63). This effect was attributed to the reduced production of the polysaccharide intercellular adhesin resulting in reduced bacterial cell adhesion, but the expression of many of the *S. aureus* genes implicated in biofilm formation, including *sarA*, was also reduced. This report employed a biofilm assay in which the medium was supplemented with glucose but not NaCl, and it did not include plasma coating. Thus, it is not possible to know how CY-158-11 compares to telithromycin or any other potential biofilm inhibitor. This illustrates the need to adopt a standardized experimental approach akin to MIC testing to allow results to be compared across compounds and laboratories so that the most effective compounds can be identified and prioritized for further study. For the reasons detailed above in the context of our studies with *S. aureus* regulatory mutants, we believe the experimental conditions we describe are appropriate for that purpose.

## MATERIALS AND METHODS

### Inhibition of biofilm formation

The 19 compounds evaluated in these studies are listed in Table 1 along with their commercial source, a literature reference relevant to their inclusion in these studies, and a brief description of the biofilm assay and bacterial strain(s) used in the cited report. Comparisons were done using the microtiter plate biofilm assay we previously described (11). Briefly, the test strain was grown overnight in tryptic soy broth (TSB) with 0.5% glucose and 3% NaCl (BFM) and then diluted to an optical density (OD_560_) of 0.05 in BFM. A 180-µL volume of the culture was added to a non-tissue culture-treated 96-well microtiter plate that had been coated overnight at 4°C with 20% human plasma in carbonate buffer. Test compounds were dissolved in DMSO at a stock concentration of 5 mM before preparing a series of twofold dilutions in DMSO. A 20-µL volume of each dilution was added to each well to achieve final concentrations ranging from 250 to 0.015 µM. This corresponds to 112.64–0.007 and 203.00–0.012 µg/mL of celastrol and telithromycin, respectively (Table 3).

**TABLE 3:** Concentrations of celastrol and telithromycin tested^*a*^

Molar concentration (µM)	Celastrol (µg/mL)	Telithromycin (µg/mL)
250	112.64	203.04
125	56.32	101.52
62.5	28.16	50.76
31.25	14.08	25.38
15.63	7.04	12.69
7.81	3.52	6.35
3.91	1.78	3.17
1.95	0.89	1.59
0.98	0.45	0.78
0.49	0.23	0.40
0.25	0.11	0.20
0.12	0.06	0.10
0.06	0.03	0.05
0.03	0.014	0.025
0.015	0.007	0.012

^*a*^ Celastrol and telithromycin were dissolved in DMSO as a 5,000-µM stock and used to make twofold dilutions. The molarity of each dilution tested is shown along with the corresponding concentrations of celastrol and telithromycin.

Plates were incubated statically overnight at 37°C. After washing, biofilms were stained with crystal violet before eluting with ethanol and measuring the absorbance at 595 nm (OD_595_) on a FLUOstar Omega plate reader (BMG Labtech). The parent strain and its isogenic *sarA* mutant were used as positive and negative controls, respectively. Because compounds were dissolved in DMSO, biofilm formation was assessed with the parent strains in the presence of an equivalent concentration of DMSO. Results are presented as percent (%) biofilm formation relative to the average observed with the corresponding parent strain in the presence of DMSO but the absence of any putative inhibitor. With both LAC and UAMS-1, this average was typically an OD_595_ ≈ 3.5. The *sarA* mutants were examined without DMSO. All experiments included two biological replicates, each of which included three experimental replicates. Statistical analysis was done by one-way ANOVA with Dunnett’s correction using GraphPad Prism version 10.0.0 for Windows (GraphPad Software). Comparisons were made to the results observed with the isogenic parent strain and to those observed with the isogenic *sarA* mutant.

### Quantitative real-time PCR

Total RNA was isolated from overnight cultures of UAMS-1 and LAC grown in BFM with and without 0.49-µM telithromycin. RNA was isolated using a Qiagen RNeasy mini kit and reverse-transcribed into cDNA using the iScript cDNA synthesis kit (Bio-Rad Laboratories). RNA concentration and purity were determined using a NanoDrop spectrophotometer (ThermoScientific). Quantitative real-time polymerase chain reaction (PCR) (qRT-PCR) was performed using a QuantStudio 6 Flex PCR system, TaqMan Fast Advanced Master Mix (Applied Biosystems), 25 ng of cDNA, 900 nM of each primer, and 250 nM of the appropriate probe. The full gene expression data set is included as Table 2. The relative fold change in gene expression was calculated using *gyrA* as a control. Primers and probes used for *sarA* and *gyrA* are listed in Table 4.

**TABLE 4:** Primers and probes used for qRT-PCR and PCR

Primers and probes	
*sarA*	Forward: ACGTAATGAGCATGATGAAAGAACTGT
	Reverse: AGTTCAATTTCGTTGTTTGCTTCAGT
	Probe: FAM-TTGTTAATGCACAACAACGT-MGBNFQ
*gyrA*	Forward: GCACGTATCGTTGGTGACGTA
	Reverse: TTGGCCATCAACAAGCGGAT
	Probe: FAM-TGGGTAAATATCACCCTCAT-MGBNFQ
*cna*	Forward: CAAGCAGTTATTACACCAGACGG
	Reverse: CACCTTTTACAGTACCTTCAATACC
*lukS*	Forward: AATTGCATTGCTTTTGCTATCC
	Reverse: ATTTTGAACCATTACCTCCACC

### Planktonic growth conditions

To assess the impact of test compounds on the growth of LAC and UAMS-1, each strain was grown overnight in TSB and then diluted to an OD_560_ of 0.05 in BFM containing each test compound at the same concentrations employed in biofilm assays. Culture tubes were not coated with human plasma. After setting up the cultures, an aliquot was immediately removed and serially diluted for plate count to verify the number of colony-forming units at T0. After incubating at 37°C for 16 h with constant shaking, the number of viable cells was determined by serial dilution and plate count. Colony counts below those observed at T0 were considered indicative of growth inhibition.

### Binding of telithromycin to SarA

The GloMelt Thermal Shift Protein Stability Kit (Biotium) was used to determine the thermal stability of SarA with or without telithromycin. A 20-µL reaction mixture was prepared in buffer (25 mM Tris, pH 7.4; 100 mM NaCl; and 0.1% NP40) containing 200 µM *SarA,* 1× GloMelt dye, and 4 mM telithromycin. The control reaction contained the same concentration of DMSO without telithromycin. Reaction mixtures were analyzed in a thermocycler (QuantStudio 6 Pro) using melt curve settings as follows: initial hold: 25°C, melt curve temp: 25°C–99°C, and ramp rate: 0.05°C/s. Data acquisition was done using a SYBR Green filter set with excitation and emission wavelengths of 470 and 520 nm, respectively. Analysis of Tm was performed using the Boltzmann equation from a plot of fluorescence intensity vs temperature in OriginPro software.

### Protease production and SDS-PAGE profile

UAMS-1 and LAC were grown overnight (16 h) in BFM with and without telithromycin at 0.49 µM. This concentration was chosen because it was the lowest concentration that inhibited biofilm formation to a degree comparable to that observed with a *sarA* mutant without limiting growth. The optical density of the culture (OD_560_) was then determined and used to adjust all cultures to an equal density. Cells were removed by centrifugation, and the resulting CM was sterilized by filtration. Total protease activity was assessed using the EnzChek gelatinase/collagenase assay kit (Thermo Fisher Scientific) after 1- and 24-h incubation (18). Because the increased protease production observed in *sarA* mutants has been correlated with the reduced abundance of *S. aureus* proteins in CM, overall protein profiles were also examined by SDS-PAGE (18, 64). Briefly, equivalent amounts of CM were examined using 4%–12% Novex Bis-Tris Plus gradient gels (Life Technologies). Proteins were visualized by staining with SimplyBlue SafeStain (Life Technologies) and imaged using the Bio-Rad ChemiDoc MP Imaging System (Bio-Rad Laboratories, Inc.).

### Autolysis assay

Overnight cultures were grown in BFM as previously described with modification (38, 65). Autolysis was assessed by the dilution of BFM overnight cultures of the designated strain to an OD_600_ of 0.05 in TSB with 1-M NaCl. Treated cultures also contained 0.12 µM or 0.49 µM of telithromycin. The control parental strain culture included 1-M NaCl and an equal concentration of DMSO as the treatment conditions. Diluted cultures were incubated for 3 h at 37°C with shaking; then, the cells were washed twice with ice-cold water and resuspended at an OD_600_ < 1.0 in 50-mM Tris-HCl with HCl with 0.05% (vol/vol) Triton X-100. A volume of 100 µL of the cell suspensions was plated in a 96-well round-bottom microtiter plate. The plate was incubated for 4 h at 30°C with shaking, and OD_580_ was measured in 30-min intervals on a FLUOstar Omega plate reader (BMG Labtech). Results are represented as % autolysis, calculated as 1 − OD_580_ at each time point divided by OD_580_ at time 0. Statistical analysis was done by one-way ANOVA with Dunnett’s correction using GraphPad Prism version 10.0.0 for Windows (GraphPad Software).

### Eradication of an established biofilm

To assess whether telithromycin has an impact on an established biofilm, we carried out experiments in which biofilms were allowed to form overnight with UAMS-1 and LAC in the absence of telithromycin. The BFM was then removed and replaced with BFM containing increasing concentrations of telithromycin. Incubation was continued overnight before assessing the amount of biofilm remaining by OD_595_ on a FLUOstar Omega plate reader (BMG Labtech). Statistical analysis was done by one-way ANOVA with Dunnett’s correction using GraphPad Prism version 10.0.0 for Windows (GraphPad Software).

### Availability of materials and culture authentication

The sources of the compounds tested in this report are listed in Table 1. Cultures of UAMS-1 (ATCC strain 49230) and LAC (kindly provided by Dr. Alex Horswill, University of Colorado School of Medicine) were maintained at −80°C and authenticated by PCR for *lukS*, a component of the Panton–Valentine leucocidin that is present in LAC but not UAMS-1, and *cna*, which encodes a collagen-binding adhesin and is present in UAMS-1 but not LAC. Primer sequences are listed in Table 4.
